# Mixing Is Dispensable for Optical Density-Based High-Throughput Growth Screening Assay in Fission Yeast

**DOI:** 10.3390/ijms27083410

**Published:** 2026-04-10

**Authors:** Kim Kiat Lim, Jiunn Jye Chung, Sha Ma, Ching-Chiuan Yen, Louxin Zhang, Ee Sin Chen

**Affiliations:** 1 Department of Biochemistry, Yong Loo Lin School of Medicine, National University of Singapore, Singapore 117596, Singapore; 2 NUS Centre for Additive Manufacturing, National University of Singapore, Singapore 117575, Singapore; mcjj@nus.edu.sg (J.J.C.); masha@nus.edu.sg (S.M.); didyc@nus.edu.sg (C.-C.Y.); 3 Department of Mathematics, Faculty of Science, National University of Singapore, Singapore 119076, Singapore; matzlx@nus.edu.sg; 4 National University Health System (NUHS), Singapore 119228, Singapore; 5 NUS Graduate School of Science & Engineering, National University of Singapore, Singapore 119077, Singapore; 6 NUS Artificial Intelligence Institute, National University of Singapore, Singapore 119391, Singapore

**Keywords:** fission yeast, *Schizosaccharomyces pombe*, microtiter plate, growth rate, doubling time, exponential phase

## Abstract

Optical density (OD)-based cell growth measurement is commonly used in high-throughput screening (HTS) during drug discovery or when deciphering the pharmaceutical mechanism of action. While resuspending the cells via a mixing step is often assumed to be necessary prior to OD measurement, its essentiality in HTS workflows has not been systematically verified. Here, through the measurement of the growth of several strains of the microbial yeast *Schizosaccharomyces pombe* cells, we compared the overall growth dynamics between samples that have been mixed and not mixed. Using statistical quantification by a two-tailed paired *t*-test followed by multiple comparison corrections, we concluded from the comparison of the doubling time of cells growing in the exponential phase that mixing did not significantly affect the biological interpretation compared to unmixed samples. Doubling time quantification between mixed and unmixed samples showed a difference of approximately 10% on average based on the assessment of the growth of eight strains. As such, if the experimental outcome can accommodate this level of variability, incorporating a mixing step before OD determination would not be necessary. These observations support the simplification of HTS processes, improving the cost efficacy and process efficiency of readouts, yet maintaining the accuracy of data acquisition.

## 1. Introduction

High-throughput screening (HTS) is a large-scale automated approach widely used in pharmaceutical or biological research focusing on drug discovery or in elucidation of gene–gene interactions. By incorporating robotic platforms, automated sample handling, coupled with suitable assays and reliable data analysis, can test a huge library of chemical compounds for biological or biochemical activities in a short period of time. Furthermore, HTS also facilitates the rapid evaluation and identification of promising novel compounds, before proceeding downstream to uncover their biological targets and the underlying mechanism of action. Alternative molecular pathways and targets can also be identified from approved drugs through HTS for drug repositioning studies and rationalizing off-target side effects [1,2,3].

A robust HTS requires four elements: (1) extensive compound libraries or samples; (2) a simple yet robust assay suitable for automation; (3) an automatic/robotic system; and (4) a systematic data handling and interpreting system [4]. To these ends, the fission yeast *Schizosaccharomyces pombe* has been widely used as a model for HTS owing to its rapid growth, ease of experimental manipulation, cost effectiveness, well annotated genome, and highly conserved physiology and genome—70.6% of the 5134 protein-coding genes show sequence similarity with that of metazoa [5]. Furthermore, this unicellular eukaryote possesses highly similar physiological pathways to humans, for example, RNA interference (RNAi) machinery, cell cycle control, DNA damage response, chromatin organization, and mitochondria function [6,7,8,9,10,11]. Its ability to grow robustly in suspension on microtiter plates or colony formation on solid agarose plates makes it versatile for large-scale phenotypic screening with diverse experimental design options [5,12]. Various kinds of screening have been carried out thus far using *S. pombe*. For example, the determination of the mechanisms of action of chemotherapeutic drugs such as doxorubicin [13,14,15,16,17] and 5-fluorouracil [18], identification of the cellular response to environmental toxins (e.g., bisphenol A) [19], identification of novel DNA damage response regulators [20,21], and the discovery of the function of previously uncharacterized genes in combination with machine learning [5].

Growth-based assays remain one of the most straightforward approaches in assessing the fitness of *S. pombe* in response to stress or chemical perturbations. Some screening methods use pins to transfer cells from the source plate onto the treatment agar plate, often incorporated with drugs to assess responsiveness, such as quantifying cellular susceptibility to various treatments through measuring the colony size [22,23]. Although this method requires minimal continuous monitoring, information can only be obtained as an endpoint readout without insights into real-time growth dynamics, which can yield valuable information on the drug effects [24,25,26,27,28,29,30,31,32]. We and others have shown previously that quantification of colony growth is prone to unresolvable edge effect errors that can severely confound the precision of the readouts, unless the procedure is extensively optimized [33,34,35].

The alternative approach to colony size measurement is the measurement of the growth rate through continuous monitoring of the OD over a time duration. This method captures the dynamics of cell growth through the capability in delineating the lag, exponential, and stationary phases of cells. Treatments usually exert their effects through slowing proliferation rather than abrupt cell death. Quantification of growth kinetics of cells, especially during the exponential phase, by measurement of growth rate and doubling time, provides a biologically interpretable endpoint that better reflects cell fitness and a chemical’s efficacy [30,31,32].

Typical HTS workflow includes sequential steps like incubation, plate handling, culture mixing, and OD measurement. All these steps are now commonly automated using a robotic platform [28,36,37,38]. Design of the automated workflow entails linking up these steps, and each additional step will lengthen handling time, increasing the risk of introducing a mechanical disturbance, which in turn leads to technical variability within the assay [39,40,41]. OD-based growth measurements can be influenced by several factors, such as the ambient temperature at the experimental site, types of containers used, media volume, and cell distribution in the media [24,42]. A mixing procedure is usually incorporated to homogenize these conditions within the samples, and this process is typically carried out using a liquid handling system that involves an automated multichannel aspiration/dispense system, particularly prior to OD acquisition [43]. A mixing step is not straightforward as it needs to ensure sterility as well as prevent cross-well contamination of samples. This stringent requirement entails dedicated set-up and the use of non-reusable consumables (tips), translating to the prolonging of time within each of the repetitive cycles and an increased operation cost. Additionally, mixing introduced shear stress to cells, might create bubbles that interfere with OD reading and may even result in sample loss due to the retention of culture within the tips [43,44,45]. A non-contact mixing method using an orbital shaker for a microtiter plate is sometimes employed, but this offers inefficient mixing and may cause cross-contamination between wells [46]. Therefore, a better substitute is needed.

The effectiveness of the mixing step has seldom been systematically evaluated in the determination of cell growth kinetics. In this study, we attempt to address this gap by comparing the growth curves between mixed and unmixed cells across multiple strains and replicates. We developed a cheaper mixing device for experimental assays that require mixing, and we showed that the growth kinetic for samples mixed with the mixer were highly comparable with those mixed with a pipette. Surprisingly, we noticed that there is no statistical difference between the growth curves of mixed and unmixed samples. The comparison of doubling time between treatments also showed no statistical differences, pointing to the possibility of omitting this step from the HTS. Together, this study provides evidence that mixing can be omitted in *S. pombe* OD-based HTS workflow.

## 2. Results

To find out whether incorporation of the mixing step before growth data acquisition via OD measurement is essential for a high-throughput long-duration growth assay, we set out to compare the cell growth in various forms: without any mixing (no mixing), mixing with a pipette (pipette), and mixing with a custom-made apparatus (mixer). Manual pipetting by drawing liquid cultures up and down a pipette tip is the standard procedure of use in low-throughput experiments to ensure thorough resuspension of precipitated cells [17,18,47,48], which is thus employed as the positive control here.

### 2.1. Construction of 96-Pin Mixer

Continuous vigorous shaking of microtiter plates poses the risk of spillage of cell suspension, and the use of a mixing pad (hereafter referred to as a mixer) can enable more controlled agitation to bring about even mixing of cells that have sunk to the bottom of the wells during incubation. Normal experimental procedure achieves this aim by manually pipetting (aspirating and dispensing) cell cultures repeatedly until they are evenly mixed. Although it can lead to highly homogenized cultures, pipetting is impractical for high-throughput workflows due to the requirement of costly liquid handling. Unmonitored mixing via pipetting may also lead to unintentional spillage during the mechanical perturbation. As such, we observe that the use of a mixer would be a compromise to achieve efficient and fast suspension of cells.

**Figure 1:**
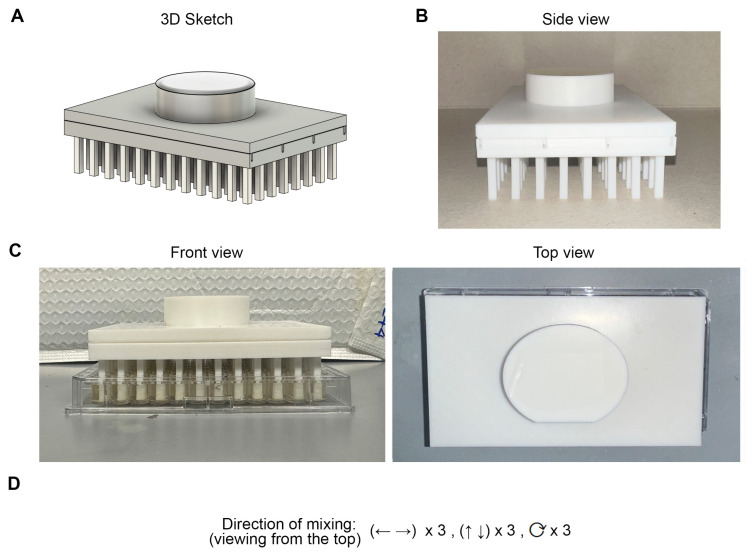
Fabrication of a 96-pin mixer for resuspending cell cultures. (**A**) 3D sketch of the mixer. (**B**) Side view of the actual 3D printed mixer. (**C**) Images showing front and top views when the mixing was performed using a mixer. (**D**) Direction of mixing if viewing from the top. It starts from three times left and right, then three times up and down, followed by three rounds of circular clockwise mixing.

We employed 96-well microtiter plates in our study here, partly due to the ease of visual monitoring of the cell cultures. We designed and fabricated a 96-pin mixer (Figure 1A) with thermoplastic polyurethane (TPU) using three-dimensional (3D) printing (Figure 1B). TPU was chosen based on its flexibility and elasticity, with good resistance against impact and abrasion, in addition to the desired hardiness of the material.

### 2.2. Procedure for Sterilization of the Mixer Apparatus

The mixer apparatus was sterilized through a series of steps before use by immersing in 70% ethanol for 5 s (s) with gentle agitation, followed by 5 s of gentle agitation in sterilized water before being tapped dry on a paper towel. An experiment was designed to ensure this sterilization process works for *S. pombe*. A fixed concentration of WT and mutant cell cultures was used to fill the columns of a microtiter plate labeled “original” (Supplementary Figure S1A). First, the sterilized mixer was used for resuspending the cultures of the “original” plate, followed by direct mixing into another microtiter plate with YEA media filled at the same column positions. This plate, which was labeled “non-sterilized”, acted as a control to show the presence of cells carried over if no sterilization was done after resuspension of the culture (Supplementary Figure S1B). Alternatively, the mixer passed through a sterilization procedure after mixing the “original” plate before mixing into another YEA-filled microtiter plate labeled “sterilized” (Supplementary Figure S1C). A spot assay was done by spotting cultures from the three labeled plates. No growth was observed for the culture obtained from the “sterilized” plate, indicating that the sterilization procedure was effective (Supplementary Figure S2). This is further supported by the absorbance reading of the plates taken before and after 21 h of incubation at 26 °C. After incubation, the OD for columns in the “sterilized” plates remained negligible, similar to that at the start of the experiment, suggesting no cross-contamination from other plates (Supplementary Figure S3).

### 2.3. Procedure for Resuspending Cultures

A 96-well microtiter plate filled with 200 μL of cell culture per well was prepared. The plate was set in a 26 °C incubator for 1 h to simulate the interval between the measurements in the actual automated workflow. To begin mixing, the sterilized mixer was placed in the plates as shown in Figure 1C. The cultures in the wells were agitated by moving the mixer three times in each of the left–right, up–down, and clockwise directions (Figure 1D; Supplementary Video S1). We ensured that the pins of the mixer touched the bottom of the wells of the microtiter plate. Thereafter, the microtiter plate was placed into the plate reader for OD acquisition.

### 2.4. Time-Course Analysis of ln(OD) Across Strains and Treatments

Eight fission yeast strains—wildtype (WT), Δ*ssb3*, Δ*dad2*, Δ*rhp55*, Δ*arp42*, Δ*apl6*, Δ*clr5* and Δ*erd2*—previously used in multidrug resistance and edge effect testing [17,33] were chosen. The mutants encompass genes that encode for proteins of various pathways: Ssb3, DNA replication protein A [49]; Dad2, a subunit of microtubule- and kinetochore-associated DASH complex [50]; Rhp55, a homologous recombination (HR) protein [51]; Arp42, a subunit of SWI/SNF chromatin remodeler [52]; Apl6, AP-3 adaptor complex subunit [53]; Clr5, a histone deacetylase [54]; and Erd2, a transmembrane receptor [55].

The growth pattern of WT and mutants was followed over a duration of 45 h to obtain growth dynamic readouts from cultures in plates treated with pipetting and mixed with the mixer apparatus relative to no mixing. Pre-cultures were prepared by growing the cells overnight in rich media. The cell cultures were diluted the following day with fresh media to an OD = 0.05, and this was designated as time = 0 h to standardize the growth progression. OD was acquired from 12 h to 24 h, with an interval of 2 h, then remeasured from 35 to 45 h. OD measurements from 25 to 34 h were omitted as preliminary experiments showed this interval corresponds to late exponential or stationary phases, which did not affect growth rate estimation. This workflow also skipped the lag phase of cells (0–12 h) to focus on the exponential phase, in which the cells attained the most optimal growth.

**Figure 2:**
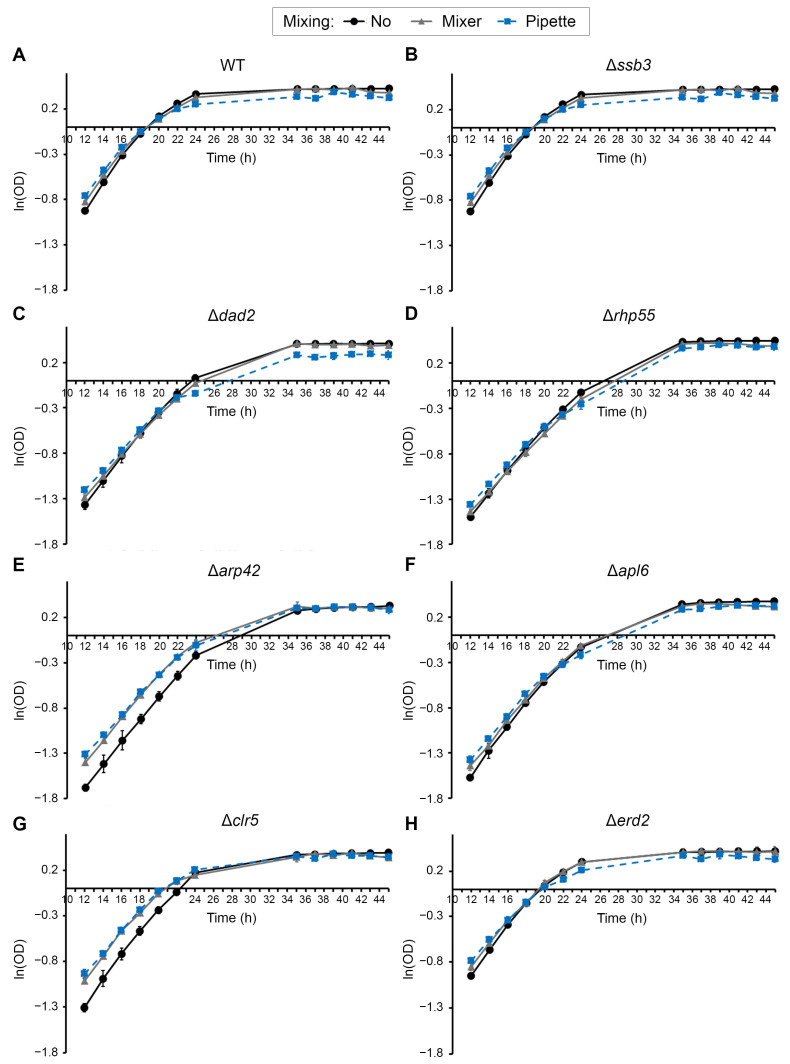
Growth curves of WT and mutants over time. Graph of ln(OD) vs. time (hour) was plotted for (**A**) WT and knockout mutants (Δ) of (**B**) *ssb3*, (**C**) *dad2*, (**D**) *rhp55*, (**E**) *arp42*, (**F**) *apl6*, (**G**) *clr5*, and (**H**) *erd2*. The results shown are the mean of three trials. Error bars represent mean ± SD, *N* = 3. Black solid line, no mixing; gray solid line, mixing with mixer; blue dashed line, mixing with pipette. Graphs of individual trials are shown in Supplementary Figure S4.

Growth curves for the aforementioned eight strains were monitored by measuring the average ln(OD) over time across three independent experimental trials (Figure 2A–H). Each trial comprised three biological replicates, with three technical replicates per biological replicate, totaling nine replicates per condition. The natural-log-transformed OD values exhibited clear linear regions corresponding to exponential growth, enabling reliable comparison of growth kinetics based on exponential growth rate and doubling time across conditions. For all strains tested, growth trajectories were highly consistent between trials, with growth curves showing a linear slope at 12–20 h, which corresponded to the exponential phase and subsequently plateaued off after 24 h when cells entered the stationary phase, demonstrating good reproducibility of the time-course measurements (Supplementary Figure S4).

The WT strain grew the fastest under all three treatments compared to the mutants, as its ln(OD) curve already crossed the horizontal time axis by 20 h (Figure 2A), followed by *ssb3* and *erd2* mutants that did so at the 20 h time point (Figure 2B,H). Among the strains tested, plots of *arp42* and *clr5* mutants repeatedly showed different growth kinetics during the exponential phase. The curves corresponding to both pipette- and mixer-mixed samples of these two mutants were located further from the non-mixed sample, showing a different trend from that of the other strains (Figure 2E,G). However, both conditions reached comparable ln(OD) levels at the stationary phase, suggesting that mixing affects growth rate without altering final biomass accumulation. The high degree of reproducibility between trials for each of the strains established the robustness of the growth measurements and provided the basis for subsequent paired statistical analyses of the time-course of growth rate and doubling time determination.

### 2.5. No Significant Difference Between Mixed and Unmixed Treatments

To determine the effect of mixing on cell growth, statistical analysis of paired time-course measurements was carried out to compare the growth curves between the treatments (no mix vs. mixer, no mix vs. pipetting, mixer vs. pipetting). The within-experiment paired difference was calculated for each time point. The values obtained for all the time points (from 12 h to 45 h) were averaged for each pair to yield a single value per trial prior to hypothesis testing (of whether the two treatments in each of the three pairs—no mix vs. mixer, no mix vs. pipetting, mixer vs. pipetting—bore a statistical difference from each other). These values from three independent biological trials were then analyzed by a two-sided paired *t*-test based on Student’s *t*-distribution, yielding one *p*-value per treatment comparison. The resulting *p*-values were adjusted for multiple comparisons using the Benjamini–Hochberg false discovery rate (BH-FDR) procedure, and statistical significance was defined based on adjusted *p*-values (*q*). Although minor variation was observed at individual time points, the composite paired time-course analyses showed no statistically significant difference between these treatments (*q* > 0.05), indicating that non-mixed samples have comparable overall growth kinetics as mixed samples with both mixer and pipetting (Table 1). In other words, the results show that mixing is dispensable in the growth curve determination assay.

### 2.6. No Statistical Difference in Doubling Time Between Treatments

Growth rate and doubling time are two disparate biological parameters to describe growth kinetics. Unlike single-time-point measurements, growth rate over a duration of time captures the proliferation capacity of cells. The growth rate is determined in the log phase when the cells divide exponentially, and represents the phase of highest sensitivity towards treatments that affect cellular fitness [56], and therefore is the readout obtained to analyze the biologically relevant outcomes of large-scale screening [28,57,58].

**Figure 3:**
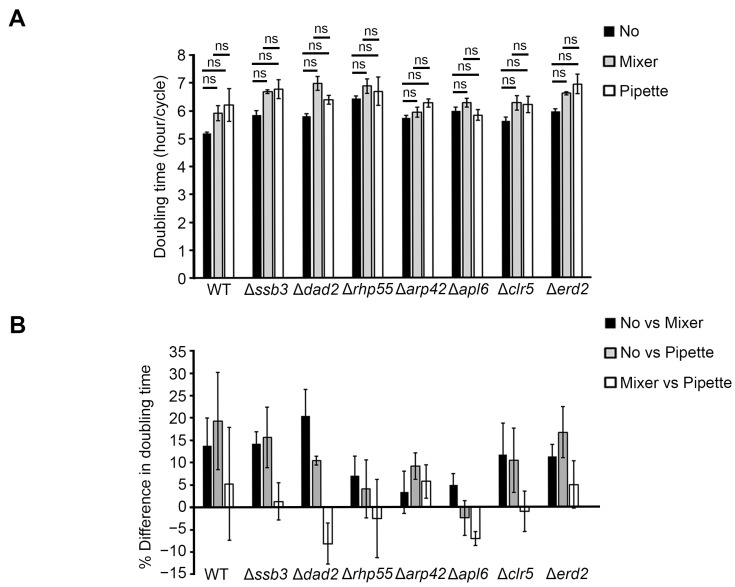
Statistically comparable doubling time between treatments. (**A**) Mean doubling time of strains under different treatment conditions: No mixing (No, black), mixing with mixer (Mixer, gray), and mixing with pipette (Pipette, white). Error bars represent mean ± SD. Statistical significance was assessed using a two-tailed paired Student’s *t*-test across trials (*N* = 3). BH-FDR correction was applied for multiple pairwise comparisons, with adjusted *p*-value (*q*) < 0.05 as significant. ns, not significant. The actual *p*-values are shown in Supplementary Table S2. (**B**) Mean percentage differences in doubling time between no mixing and mixing with mixer (black), no mixing and mixing with pipette (gray), mixing with mixer and with pipette (white). Error bars represent mean ± SD.

To assess whether mixing with a mixer or pipetting would affect the growth kinetics of cells relative to non-mixed samples, the growth rate of strains in each replicate and trial was determined from the slope of the exponential phase and then converted to doubling time. The slope was determined individually for each strain by selecting time points within the 12–20 h interval that demonstrated the strongest linearity (R^2^ ≥ 0.98). Under non-mixed conditions, WT grew the fastest with a doubling time of 5.2 ± 0.04 h/cycle, while the slowest strain—a mutant of *rhp55*—grew 1.23-fold slower than WT (Figure 3A; Supplementary Table S1). Rhp55, an HR protein, is important for DNA damage repair and resolving stalled replication forks [59,60]. The slower growth of Δ*rhp55* might be due to the inefficient HR for DNA damage arising in normal cellular processes [51]. Most strains except Δ*apl6* and Δ*rhp55* showed increased doubling time for mixed compared to non-mixed samples (Figure 3A). Initial analysis suggested that a subset of strains exhibited differences in doubling time between treatments; however, adjusted *p*-values for multiple comparisons across all treatment pairs and strains using BH-FDR correction show no statistically significant differences between treatments (Figure 3A; Supplementary Tables S1 and S2). The observed variations in doubling time are likely due to experimental variability rather than treatment-specific effects.

Further comparison between treatments shows that the percentage difference in doubling time for mixer vs. non-mixed samples ranged from 3.3 to 20.4%, pipette-mixed to non-mixed samples ranged from −2.5 to 19.3%, while mixing with pipette to mixer ranged from −8.2 to 5.72% (Figure 3B; Supplementary Table S3). Mean percentage difference for mixer- and pipette-mixed samples with non-mixed samples for the eight strains tested were 10.8 ± 5.6% and 10.4 ± 7.1%, respectively (Supplementary Table S3). Analysis also showed that mixing with a mixer and a pipette was closely similar, with only a −0.23 ± 5.49% difference in doubling time. Therefore, in an experimental context where a ~10% variation in doubling time is biologically acceptable, the omission of the mixing steps from the procedure will likely not affect the downstream decision-making and result interpretation.

### 2.7. Mixing Does Not Affect the Cell Biology and Cell Viability

To assess whether the observed variation in doubling time and growth curves between different treatments could be due to the effect of mixing on cell biology or viability, an experiment was designed. During the incubation period at 12–20 h, microtiter plates with cells were either mixed or not mixed every two hours (Supplementary Figure S5A). At the 20th h, cells were glutaraldehyde-fixed and cell cycle distribution was quantified by scoring the proportion of cells at different stages, while cell viability was assessed using phloxine B staining. In the control strain, the temperature-sensitive mutant of Cnp1 (*cnp1-1*) exhibited a decrease in viability upon shifting to a higher temperature, consistent with previous reports, as indicated by an increase in cells stained with phloxine B (Supplementary Figure S5B) [61]. However, the number of phloxine B-stained cells did not increase in all the mutants and WT tested under all treatment conditions, suggesting that mixing does not affect the cell viability (Supplementary Figure S5C). Next, scoring of the glutaraldehyde-fixed cells shows no obvious changes in the cell cycle distribution for all the strains tested, suggesting that mixing has no impact on cell biology (Supplementary Figure S5D).

## 3. Discussion

HTS has been widely applied across diverse organisms, including mammalian cells, yeast, and bacteria [30,56,62], and to a broad range of applications such as profiling of drug sensitivity, genetic interactions mapping to pathway discovery, and analysis of environmental response [5,31]. Nevertheless, ongoing efforts are carried out to refine HTS workflows to enhance efficiency, robustness, and scalability [33,36,37,63]. In this study, we systematically assess the essentiality of cell culture mixing in the HTS workflow and its influence on growth kinetics, employing *S. pombe* as a model organism. We found that mixing is dispensable for OD-based HTS during the comparison of overall growth of cells, or when the experiment can tolerate a variation of ~10% in doubling time determination.

### 3.1. Interpretation of ~10% Variability in Doubling Time and Ways to Improve Assay Sensitivity

Percentage-based normalization can amplify relatively small absolute differences, which explains the broad range of percentage differences in doubling time observed across strains (Supplementary Table S3). However, statistical comparisons, followed by BH-FDR correction on the raw doubling time value detected no significant differences between treatments across all strains tested, indicating that the mixing step does not incur a reproducible effect on the doubling time. The biological relevance of the approximately 10% variation in doubling time between mixed and non-mixed samples depends strongly on the context of screening. This variation is less likely to affect screens designed to identify strong growth defects, but more so for screens that aim to detect subtle drug responses or modest growth defects. A ~10% variation could reduce the assay sensitivity and might obscure weak but biologically meaningful effects. In this case, assay sensitivity could be increased, for example, by using strains with a sensitized genetic background such as mutants defective in multidrug resistance pathways, or by combining mutations (e.g., double mutants) to uncover additive or synergistic effects on drug sensitivity [64].

### 3.2. Shaking or Mixing Is Not Necessary for Time-Course OD Measurements

It is intuitively assumed that mixing of cell cultures in microtiter plates presumably creates a homogenized suspension for accurate measurement of OD. It is therefore unsuspected that mixing did not significantly increase the accuracy over unmixed samples in terms of growth rate and doubling time measurements.

We noted a valuable study that also reported that continuous or intermittent shaking of microtiter plates during HTS can even lead to cell clumping at the center and periphery of wells, thereby confounding and increasing variability in OD measurements [56]. They interestingly show that increasing concentration of the starting cultures can surprisingly promote the formation of a uniform cell lawn to mitigate concerns related to oxygen and nutrient availability, which otherwise will lead to cell aggregation, especially in lower volumes and inoculum sizes [56]. In a way, our findings concur with this study by positing that mixing is not required, while that study went on to say that mixing can artificially introduce artifacts.

Furthermore, many studies that employed microtiter plate-based growth studies have relied on orbital shaking with either standalone shakers or a shaker built into a plate-reader to homogenize cell suspensions and facilitate nutrient and oxygen distribution [42,65]. Many even went to an extra extent to devise distinct shaking patterns, either continuous or intermittent shaking, various shaking speeds, directions, and duty cycles [28,31,66]. Such interventions often require additional hardware, including dedicated plate readers for prolonged continuous OD monitoring or even multiple instruments operating concurrently [31]. Therefore, the shaking/mixing step constitutes a bottleneck in the HTS workflow to restrict scalability and increase experimental complexity. In this context, the recommendation from our findings will reduce experimental complexity, free up hardware (such as plate reader equipment), and sidestep the need to optimize assay-specific shaking parameters.

### 3.3. Usefulness of a 96-Pin Mixing Pad When Mixing Is Required

One exception to the applicability of a mixing-free workflow is when using strains prone to aggregate or form filaments, which greatly augments the speed at which these cells sink to the bottom of the wells, and continuous vigorous agitation is required to achieve adequate cell dispersion. For example, mutants of a fission yeast transcription factor, such as Δ*rfl1*, flocculate easily, while those of pentatricopeptide repeat (PPR) protein (e.g., *ppr3*, *ppr4*, *ppr6*, and *ppr10*) also flocculate and further form filamentous mass [67,68]. For these strains, mixing becomes critical to promote cell dispersion before OD measurement. Orbital shaking is, however, less efficient than magnetic bar stirring as the even dispersion highly depends on well geometry and rotation conditions [46]. As it is impractical to put 96 magnetic stirrers inside the wells, the mixer pin tools we have reported here will be an alternative, especially when we showed that the mixing efficacy is equivalent between the use of the mixer apparatus and pipetting (Figure 3B; Supplementary Table S3).

Mixing or not is context dependent. For example, in the study of biofilm formation, mixing should be excluded from the workflow. If the growth conditions differ from those described here—for example, when using minimal media instead of rich YEA medium, different incubation temperatures, or using species other than *S. pombe*—pilot experiments should be performed to assess the necessity of mixing. This is because different species have different sedimentation properties, growth rates, and clumping tendencies. Furthermore, if the determination of doubling time is the primary objective, preliminary testing of the strains of interest should be conducted prior to large-scale experiments to identify strains that require mixing. In this way, our study provides guidance for both scenarios—when mixing is required and when it can be omitted—to aid in the robustness of microtiter plate-based HTS workflow.

### 3.4. Benefits and Caveats of Liquid-Based over Agar-Based Assays

In our study, we obtained reproducible growth curves encompassing both exponential and stationary phases of the cell for different trials (Figure 2). Growth kinetics of cells were determined from the exponential phase of ln(OD) versus time plots, while differences in overall growth behavior, including biomass accumulation and entry into the stationary phase, were evaluated by paired time-course analysis (Figure 3A; Table 1). This method of assessing growth fitness of cells is quantitative, less subjective, and higher throughput over the conventional drug or chemical testing in yeast, which commonly employs spot assays as a means of assessing growth sensitivity and viability under stress conditions [13,69]. Furthermore, the availability of software such as GrowthRates (ver. 6.2.1, https://sourceforge.net/projects/growthrates/files/ (accessed on 6 April 2026)), GATHODE (Growth Analysis Tool for High-throughput Optical Density Experiments, https://platereader.github.io/index.html (accessed on 6 April 2026)), SPOCK (https://github.com/labmccormick/SPOCK (accessed on 6 April 2026)), and PRECOG (PREsentation and Characterization Of Growth-data, http://precog.lundberg.gu.se/Pages/Content/GettingStarted (accessed on 6 April 2026)) has facilitated the quantitative assessment of cell fitness more readily [25,28,70,71,72].

Spot assays and endpoint OD measurements provide only cumulative outcomes that integrate all growth phases, without providing information about underlying growth dynamics [19,34]. Although significant advancement have been made to increase the throughput and accuracy—for example, using pin tools for high-throughput screening [5,73], analyzing the spot growth quantitatively [74], or incorporating time-course imaging to approximate growth dynamic rather than relying solely on static endpoints [23,34]—assaying colonies on agar media still entails considerably longer time durations and provides less informative than liquid-based assay. Although agar growth analysis has seen significant advancements through assistance by microscopy, which improves sensitivity and matches the timeframe of liquid assays, the method is still not well suited for HTS or automation due to limited handling flexibility and complex setup requirements, e.g., humidified chambers and microscopy systems [75,76].

Analysis of cell fitness in liquid cultures is capable of capturing small phenotypic changes in different phases of cells and requires far fewer chemicals than the spot assay [56]. However, some precautions need to be considered when using a microtiter plate. Although overall cell growth and global protein expression remain largely unchanged in microcultures, modest differences have been reported in the expression of proteins associated with stress responses and oxygen limitation [58,66]. This explained why the doubling time we obtained, even for wildtype cells, ranged from 5.2 to 6.2 h/cycle in different treatments, different from what is usually reported, which is 3.5–4 h (Supplementary Table S1) [6,77]. Nonetheless, such a microculture-associated effect does not negate the use of microtiter plate-based assays, which still remains the mainstay of HTS owing to its consistency and reproducibility, ease of use, and low cost.

## 4. Materials and Methods

### 4.1. Experimental Design

Nine replicates of cells actively growing in YEA media (3% glucose, 0.5% yeast extract, 75 mg/L adenine), consisting of wildtype (WT) and mutants with one of the following gene-knockout (Δ) at a time: *ssb3*, *dad2*, *rhp55*, *apl6*, *arp42*, *clr5*, and *erd2*, obtained from Nguyen et al. [17], were diluted to OD_600_ = 0.05. A total of 200 μL of cell culture for each strain was placed inside separate wells of the 96-well microtiter plate (Greiner Bio-One, sterile F-bottom with lid, Cat. No. 655180; Kremsmünster, Austria). Three similar plates with cells were prepared and labeled as no mixing (‘No mix’), mix with mixer (‘mixer’, a custom-made 96-pin mixing pad), and mix with pipette (‘pipette’).

Microtiter plates were then placed inside a sealed container containing water-soaked paper towels, creating a custom humidified chamber, before placing them into 26 °C incubator. OD for the plates was taken using a BioTek Synergy H1 microplate reader (Agilent; Santa Clara, CA, USA) at 600 nm wavelength with a 2 h interval starting from 12 to 24 h and then 35 to 45 h. Before the OD was taken, the plates were removed from the incubator, and mixing was done using the mixer (Figure 1) or a pipette. For the mixer, it was first dipped into 70% ethanol for 5 s with gentle agitation to sterilize the pins, then dipped inside sterile water for 5 s with gentle agitation to remove excess ethanol, following by drying the pins by repeated tapping the mixer on clean Kimwipes (Kimberly-Clark Professional; Roswell, GA, USA) until there was no visible water mark on the latter. The mixer was inserted into the microtiter plate labeled ‘mixer’, doing the following motion in order: moving up and down three times, left and right three times, and clockwise circular three times (Figure 1C,D; Supplementary Video S1). For pipette mixing, the wells of the plate labeled ‘pipette’ were mixed by pipetting up and down gently three times using a multichannel pipette, without generating bubbles. The whole experiment was repeated twice independently.

### 4.2. Verifying the Effectiveness of Sterilizing Procedure

Log-phase growing WT and mutant cells were diluted to OD_600_ = 0.4 and 0.2, respectively, and 200 μL of each was aliquoted to the wells in columns #3 and #8 of the microtiter plate. Wells at column #10 were filled with YEA media. The plate was labeled “original” (Supplementary Figure S1A). A sterilized mixer tool was first used to mix the “original” plate, then subsequently mix the “non-sterilized”-labeled plate filled with YEA media. This “non-sterilized” plate acted as a control to show the presence of cells carried over if no sterilization procedure was carried out (Supplementary Figure S1B). To check the effectiveness of the sterilization procedure, the sterilized mixer was first used to mix the “original” plate, then subjected to the sterilization steps—5 s in 70% ethanol with agitation, 5 s in sterile water with agitation and tapped dry on Kimwipes, before mixing the “sterilized”-labeled plate filled with YEA (Supplementary Figure S1C). A total of 3 μL of cells from each plate were spotted on a YEA agar plate and incubated at 30 °C for three days before growth was recorded. An OD reading of these microtiter plates was taken before and after 21 h incubation at 26 °C.

### 4.3. Microscopic Observation

For phloxine B staining of cells, 5 μg/mL of phloxine B (Nacalai Tesque Inc.; Kyoto, Japan) was added to the cell culture and incubated for 1 h. The cells were washed thrice with 1× PBS before observation under DIC and TRITC channels of Nikon Eclipse Ti-E fluorescence microscope (Nikon; Tokyo, Japan). For cell cycle distribution analysis, cells were fixed with 5% glutaraldehyde (Sigma-Aldrich; St. Louis, MO, USA) for 15 min on ice before being washed thrice with 1× PBS. Nuclear morphology was observed under a microscope after staining the cells with 4′,6-diamidino-2-phenylindole (DAPI) (Thermo Fisher Scientific; Waltham, MA, USA).

### 4.4. Data Transformation, Growth Curve Plotting, Determination of Growth Rate, and Doubling Time

The OD data obtained were first natural-log transformed [ln(OD)] prior to graph plotting [ln(OD) versus time] (Figure 2) or proceeded with subsequent statistical analysis below. From the plotted graph, growth rate (μ, in units of *h*^−1^) for each replicate corresponded to the slope of the best-fit line estimated from the ln(OD) values during the exponential growth phase (~12–20 h). Doubling time (*t_d_*), in units of hours per cycle, was calculated as follows:

$$t_{d}=\;\frac{ln(2)}{\mu }$$

For direct comparison between treatments, percentage differences in doubling time between treatments (%${\Delta t}_{d}$) were calculated relative to the reference condition ($t_{d.reference}$), for which the equation is shown below. Positive values indicate an increase in doubling time (slower growth), while negative values indicate the opposite.

$$\%\;∆t_{d}=\frac{t_{d,treatment}-t_{d,reference}}{t_{d,reference}}\times 100$$

### 4.5. Statistical Analysis of Paired Time-Course Measurements

There are 9 replicates in each trial, a total of three trials. Measurements of OD were collected at 13 matched time points at hours 12, 14, 16, 18, 20, 22, 24, 35, 37, 39, 41, 43, and 45. To assess whether treatment—mixing with the mixer or pipette—altered the measured response (OD reading), within-experiment paired differences (*d*_i_(*t*)) were first computed at each time point using:

$$d_{i}\left(t\right)=a_{i,after}\left(t\right)-a_{i,before}\left(t\right),\;1\leq i\leq 9,\;1\leq t\leq 13.$$

where *a_i,after_*(*t*) represents the measurement of mixing (mixer or pipette), and *a_i,before_*(*t*) represents the measurement of no mixing.

To consider the repeated measurement over time, the mean difference across time within each experiment was calculated, resulting in a single difference (*D_i_*) per replicate and with nine independent values (D_1_, …, D_9_) per trial.

$$D_{i}=\frac{1}{13}\sum_{t=1\;}^{13}d_{i}(t)$$

The null hypothesis states that the mean difference between two treatments tested is zero ($H_{0}$:$\mu_{D}$ = 0) using a two-sided one-sample *t*-test. Here, $\mu_{D}$ is the population mean of the differences. For each biological trial, the mean difference ($\bar{D}$) across technical replicates was calculated to obtain a single trial-level effect estimate:

$${\bar{D}}_{j}=\;\frac{1}{9}\sum_{i=1\;}^{9}D_{i},\;j=1,\;2,\;3$$

where ${\bar{D}}_{j}$ represents the average treatment difference for the *j* number of biological trials. The trial-level estimates were further averaged to obtain an overall mean effect (${\bar{D}}_{bio}$) to take into account the variability across independent biological trials:

$${\bar{D}}_{bio}=\frac{{\bar{D}}_{1}+{\bar{D}}_{2}+{\bar{D}}_{3}}{3}$$

The standard deviation of these differences was calculated as:

$$S_{bio}=\;\sqrt{\frac{1}{2}\sum_{j=1}^{3}{\left({\bar{D}}_{j}-\;{\bar{D}}_{bio}\right)}^{2}}$$

The observed Test statistic ($t_{abs}$) is calculated as below, following Student’s t-distribution with 2 degrees of freedom. $n_{bio}$ is the number of pairs (sample size), which is 3 in this case. $S_{bio}$/($\sqrt{n_{bio}}$) represents the standard error of the mean difference.

$$t_{obs}=\frac{{\bar{D}}_{bio}}{S_{bio}/\sqrt{n_{bio}}}$$

Two-sided *p*-values are calculated as below. *p* is the probability that a random variable *T* drawn from a *t*-distribution with 2 degrees of freedom is greater than or equal to the absolute value of the observed *t*-statistic (|$t_{obs}$|). In Excel, the equation is “=2*(1-(*T.DIST*(*ABS*($t_{obs}$),*df*,*TRUE*))”.

$$p=2\;Pr(T_{2}\;\geq \left|t_{obs}\right|)$$

A *p*-value of <0.05 is considered statistically significant. All the formulas, including the values, *p*-values were calculated using Excel (Microsoft 365).

### 4.6. Multiple Testing Correction Using Benjamini–Hochberg False Discovery Rate (BH-FDR)

To control for multiple comparisons, *p*-values were adjusted using the BH-FDR procedure. For each comparison type (No vs. Mix, No vs. Pipette, and Mix vs. Pipette), *p*-values obtained across the eight strains were treated as a family of tests and adjusted independently. The BH-FDR procedure was performed as follows:

For a given *m* number of *p*-values:

$$p_{1},p_{2},\ldots ,p_{m}$$

The *p*-values were first sorted in increasing order and assigned a rank, starting from the smallest at number 1:

$$p_{\left(1\right)}\leq p_{\left(2\right)}\leq \cdots \leq p_{\left(m\right)}$$

For each ranked *p*-value $p_{\left(i\right)}$, a BH-adjusted value was calculated using:

$$q_{\left(i\right)}=\frac{m}{i}\cdot p_{\left(i\right)}$$

To ensure monotonicity of adjusted *p*-values, the corrected values were further adjusted such that:

$$q_{\left(i\right)}=min\left(q_{\left(i\right)},q_{\left(i+1\right)}\right)$$

applied from largest to smallest *p*-value.

The adjusted *p*-values (*q*-values) were then compared to a threshold of 0.05, with values of q<0.05 considered statistically significant.

## 5. Conclusions

This work demonstrates that OD-based growth assays yield comparable growth kinetics without mixing, with no statistically significant differences in overall growth curves and only modest variability in doubling time. These findings support the simplification of the HTS workflow, improving efficiency and scalability without compromising data interpretation.

## Figures and Tables

**Table 1:** Summary of statistical analysis of paired time-course measurements. Differences between treatments were first summarized within each biological trial (*N* = 3) and subsequently compared across trials. To account for multiple testing across all strains and comparisons (24 tests in total), *p*-values were adjusted using the BH-FDR procedure. Adjusted *p*-values (*q*) are shown alongside raw *p*-values, and significance annotations are based on BH-adjusted values. ns, not significant; No, unmixed; Mix, mixer mixed; Pipette, pipette mixed. No comparisons remained significant after BH-FDR correction.

Strain	Comparison	Raw *p*-Value	BH-Adjusted *p*-Value (*q*)	Significant (*q* < 0.05)
WT	No vs. Mix	0.667	0.890	ns
	No vs. Pipette	0.257	0.576	ns
	Mix vs. Pipette	0.054	0.216	ns
Δ*ssb3*	No vs. Mix	0.863	0.920	ns
	No vs. Pipette	0.227	0.545	ns
	Mix vs. Pipette	0.030	0.192	ns
Δ*dad2*	No vs. Mix	0.931	0.931	ns
	No vs. Pipette	0.235	0.545	ns
	Mix vs. Pipette	0.034	0.192	ns
Δ*rhp55*	No vs. Mix	0.306	0.612	ns
	No vs. Pipette	0.496	0.745	ns
	Mix vs. Pipette	0.167	0.445	ns
Δ*arp42*	No vs. Mix	0.067	0.216	ns
	No vs. Pipette	0.066	0.216	ns
	Mix vs. Pipette	0.395	0.703	ns
Δ*apl6*	No vs. Mix	0.603	0.860	ns
	No vs. Pipette	0.566	0.850	ns
	Mix vs. Pipette	0.965	0.965	ns
Δ*clr5*	No vs. Mix	0.072	0.216	ns
	No vs. Pipette	0.050	0.216	ns
	Mix vs. Pipette	0.042	0.192	ns
Δ*erd2*	No vs. Mix	0.482	0.745	ns
	No vs. Pipette	0.426	0.710	ns
	Mix vs. Pipette	0.044	0.192	ns

## Data Availability

The original contributions presented in this study are included in the article/Supplementary Materials. Further inquiries can be directed to the corresponding authors.

## Supplementary Materials

The following supporting information can be downloaded at: https://www.mdpi.com/article/10.3390/ijms27083410/s1.

## Abbreviations

The following abbreviations are used in this manuscript:

OD	Optical density
HTS	High-throughput screening
WT	Wildtype
TPU	Thermoplastic polyurethane
h	Hour
s	Second
#	Number
