# Supplementary material for: Mixing Is Dispensable for Optical Density-Based High-Throughput Growth Screening Assay in Fission Yeast
Source: Int J Mol Sci. 2026 Apr 10;27(8):3410. doi: 10.3390/ijms27083410 (PMC13115688; doi:10.3390/ijms27083410)
Supplement: Supplementary file 1 [file ijms-27-03410-s001.zip › ijms-4160497-supplementary.pdf]

SUPPLEMENTARY DATA

(3 Tables, 5 Figures and 1 Video with Legends)

Mixing is Dispensable for Optical Density-Based High-Throughput Growth Screening Assay in Fission Yeast

Kim Kiat Lim [1,*], Jiunn Jye Chung [2], Sha Ma [2], Ching-Chiuan Yen [2], Louxin Zhang [3], and Ee Sin Chen [1,4,5,6,*]

1 Department of Biochemistry, Yong Loo Lin School of Medicine, National University of Singapore, Singapore 117596, Singapore

2 NUS Centre for Additive Manufacturing, National University of Singapore, Singapore 117575, Singapore

3 Department of Mathematics, Faculty of Science, National University of Singapore, Singapore 119076, Singapore

4 National University Health System (NUHS), Singapore 119228, Singapore

5 NUS Graduate School of Science & Engineering, National University of Singapore, Singapore 119077, Singapore

6 NUS Artificial Intelligence Institute, National University of Singapore, Singapore 119391, Singapore

* Correspondence: bchlimk@nus.edu.sg (K.K.L.); bchces@nus.edu.sg (E.S.C.); Tel.: +65-6516-5616 (E.S.C.)

Supplementary Table S1. Doubling time of strains in three trials. The unit is hour per cycle. Mixer, mixing with mixer; Pipette, mixing with pipette.

	No mix					Mixer				
**Trial**	**1**	**2**	**3**	**Mean**	**S.D.**	**1**	**2**	**3**	**Mean**	**S.D.**
WT	5.16	5.20	5.24	5.20	0.04	6.19	5.91	5.64	5.91	0.27
Δ*ssb3*	5.69	5.95	5.92	5.85	0.14	6.66	6.75	6.62	6.68	0.07
Δ*dad2*	5.85	5.66	5.85	5.79	0.11	6.68	7.13	7.09	6.96	0.25
Δ*rhp55*	6.50	6.33	6.45	6.42	0.09	6.66	6.79	7.17	6.88	0.26
Δ*arp42*	5.84	5.67	5.71	5.74	0.09	5.72	6.03	6.04	5.93	0.18
Δ*apl6*	6.02	5.82	6.09	5.98	0.14	6.15	6.21	6.45	6.27	0.16
Δ*clr5*	5.66	5.72	5.47	5.62	0.13	6.25	6.02	6.52	6.26	0.25
Δ*erd2*	5.98	5.83	6.02	5.94	0.10	6.56	6.67	6.59	6.61	0.06

	Pipette				
**Trial**	**1**	**2**	**3**	**Mean**	**S.D.**
WT	5.63	6.80	6.18	6.20	0.58
Δ*ssb3*	6.87	7.04	6.39	6.76	0.34
Δ*dad2*	6.43	6.21	6.52	6.39	0.16
Δ*rhp55*	7.15	6.15	6.77	6.69	0.50
Δ*arp42*	6.24	6.13	6.42	6.26	0.15
Δ*apl6*	5.62	5.87	5.99	5.83	0.19
Δ*clr5*	5.90	6.21	6.48	6.20	0.29
Δ*erd2*	6.60	6.91	7.30	6.94	0.35

Supplementary Table S2. Pairwise comparisons of doubling time between treatments across eight *S. pombe* strains. Raw *p*-values were adjusted using the Benjamini–Hochberg false discovery rate (BH-FDR) correction applied across all comparisons (n= 24). Adjusted *p*-values (*q*-values) are reported, and comparisons with $q < 0.05$ (*) were considered statistically significant.

Strain	Comparison	Raw *p*-Value	BH-Adjusted *p*-Value (*q*)	Significant ($q < 0.05$)
WT	No vs Mix	0.060	0.192	ns
	No vs Pipette	0.091	0.218	ns
	Mix vs Pipette	0.574	0.766	ns
Δ*ssb3*	No vs Mix	0.009	0.108	ns
	No vs Pipette	0.054	0.192	ns
	Mix vs Pipette	0.649	0.812	ns
Δ*dad2*	No vs Mix	0.025	0.150	ns
	No vs Pipette	0.004	0.096	ns
	Mix vs Pipette	0.095	0.218	ns
Δ*rhp55*	No vs Mix	0.109	0.233	ns
	No vs Pipette	0.388	0.649	ns
	Mix vs Pipette	0.644	0.812	ns
Δ*arp42*	No vs Mix	0.355	0.609	ns
	No vs Pipette	0.033	0.158	ns
	Mix vs Pipette	0.112	0.233	ns
Δ*apl6*	No vs Mix	0.074	0.211	ns
	No vs Pipette	0.380	0.649	ns
	Mix vs Pipette	0.016	0.128	ns
Δ*clr5*	No vs Mix	0.098	0.218	ns
	No vs Pipette	0.124	0.248	ns
	Mix vs Pipette	0.713	0.820	ns
Δ*erd2*	No vs Mix	0.017	0.128	ns
	No vs Pipette	0.037	0.177	ns
	Mix vs Pipette	0.244	0.488	ns

Supplementary Table S3. Percentage difference in doubling time between treatments.

	No mixing vs Mixer					No mixing vs Pipette				
**Trial**	**1**	**2**	**3**	**Mean**	**S.D.**	**1**	**2**	**3**	**Mean**	**S.D.**
WT	20.03	13.73	7.61	13.79	6.21	9.17	30.79	18.01	19.32	10.87
Δ*ssb3*	17.12	13.54	11.90	14.19	2.67	20.67	18.33	7.97	15.66	6.76
Δ*dad2*	14.11	25.95	21.21	20.42	5.96	9.82	9.74	11.56	10.37	1.03
Δ*rhp55*	2.52	7.32	11.26	7.03	4.38	10.06	-2.84	4.98	4.07	6.50
Δ*arp42*	-2.14	6.25	5.77	3.29	4.72	6.76	8.07	12.49	9.11	3.01
Δ*apl6*	2.10	6.83	5.90	4.94	2.50	-6.70	0.99	-1.74	-2.49	3.90
Δ*clr5*	10.44	5.25	19.21	11.63	7.06	4.15	8.71	18.36	10.41	7.26
Δ*erd2*	9.79	14.37	9.51	11.22	2.73	10.32	18.46	21.26	16.68	5.68
**Mean**				**10.81 ± 5.59%**					**10.39 ± 7.12%**	

	Mixer vs Pipette				
**Trial**	**1**	**2**	**3**	**Mean**	**S.D.**
WT	-9.05	15.00	9.66	5.21	12.63
Δ*ssb3*	3.03	4.22	-3.51	1.25	4.16
Δ*dad2*	-3.76	-12.87	-7.96	-8.20	4.56
Δ*rhp55*	7.36	-9.47	-5.64	-2.58	8.82
Δ*arp42*	9.10	1.71	6.36	5.72	3.73
Δ*apl6*	-8.62	-5.46	-7.22	-7.10	1.58
Δ*clr5*	-5.69	3.29	-0.71	-1.04	4.50
Δ*erd2*	0.49	3.57	10.73	4.93	5.26
**Mean**				**-0.23 ± 5.49%**	

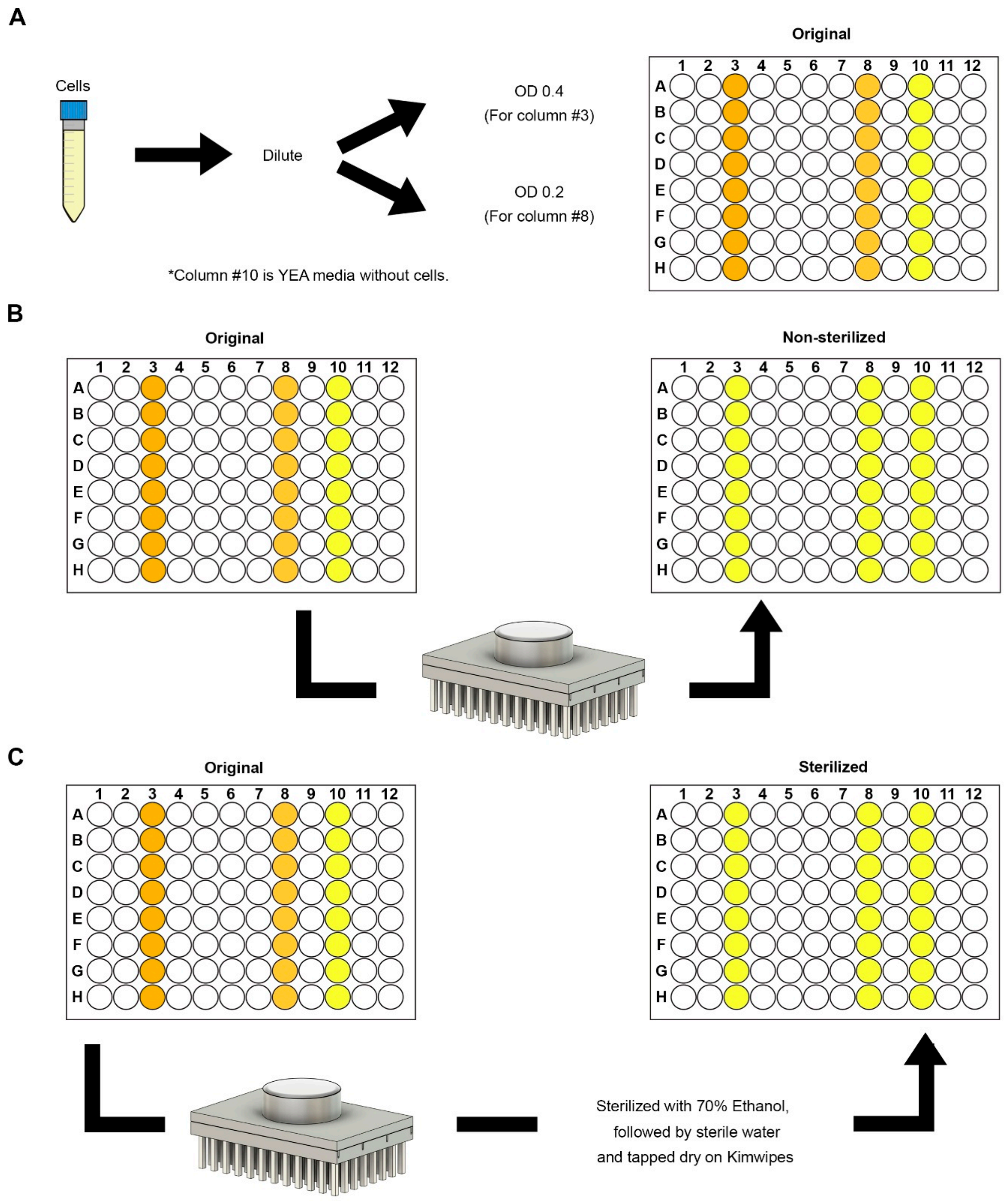

Supplementary Figure S1. Procedures to check the effectiveness of sterilization method used. (**A**) Log-phase growing cells were diluted to OD_{600} 0.4 and 0.2, then 200 µL of the cells were aliquoted to column #3 and #8 of microtiter plate respectively. Column #10 was filled with same volume of YEA media. The plate was labeled "original". (**B**) A sterilized mixer was used to mix the cell culture in the "original" plate and then directly mixed into a plate with column #3, #8 and #10 filled with YEA media. The plate was labeled "non-sterilized", acted as a control. (**C**) The "original" plate was mixed with a sterilized mixer, followed by sterilization with 70% ethanol and rinsed with sterile water. The mixer was further

tapped dry on Kimwipes before inserted into a new microtiter plate with YEA media filled at column #3, #8 and #10. The plate was labeled as "sterilized". Row A to H corresponds to strains: WT, Δ*ssb3,* Δ*dad2,* Δ*rhp55,* Δ*arp42,* Δ*apl6,* Δ*clr5* and Δ*erd2* respectively.

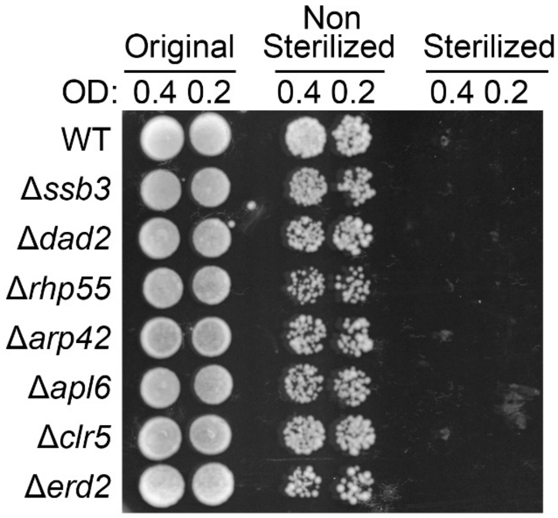

Supplementary Figure S2. Spotting assay for determination of the successfulness of sterilization procedure. 3 μL of cell culture from "original", "non-sterilized" and "sterilized" labeled plate in Supplementary Figure 1 were spotted on YEA agar plate and incubated at 30 °C for three days before plate was scanned. OD 0.4 and 0.2 correspond to column #3 and #8 of microtiter plate respectively.

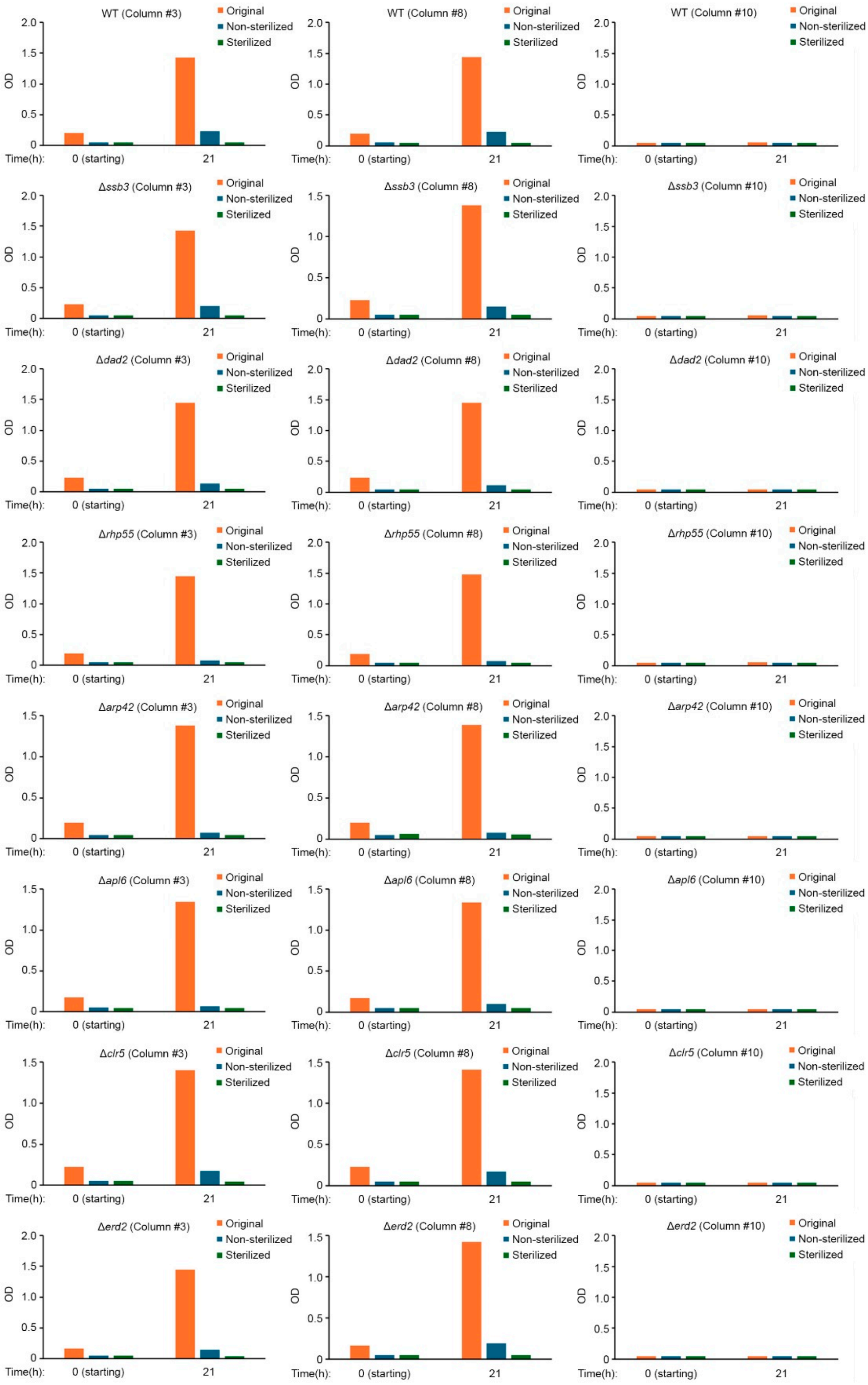

Supplementary Figure S3. Absorbance readings show successful sterilization of the mixer. OD reading of WT, Δ*ssb3*, Δ*dad2*, Δ*rhp55*, Δ*arp42*, Δ*apl6*, Δ*clr5* and Δ*erd2* present at column #3 and #8 of different plates prepared in Supplementary Figure 1, at time 0 and after the incubation at 26 °C for 21 h.

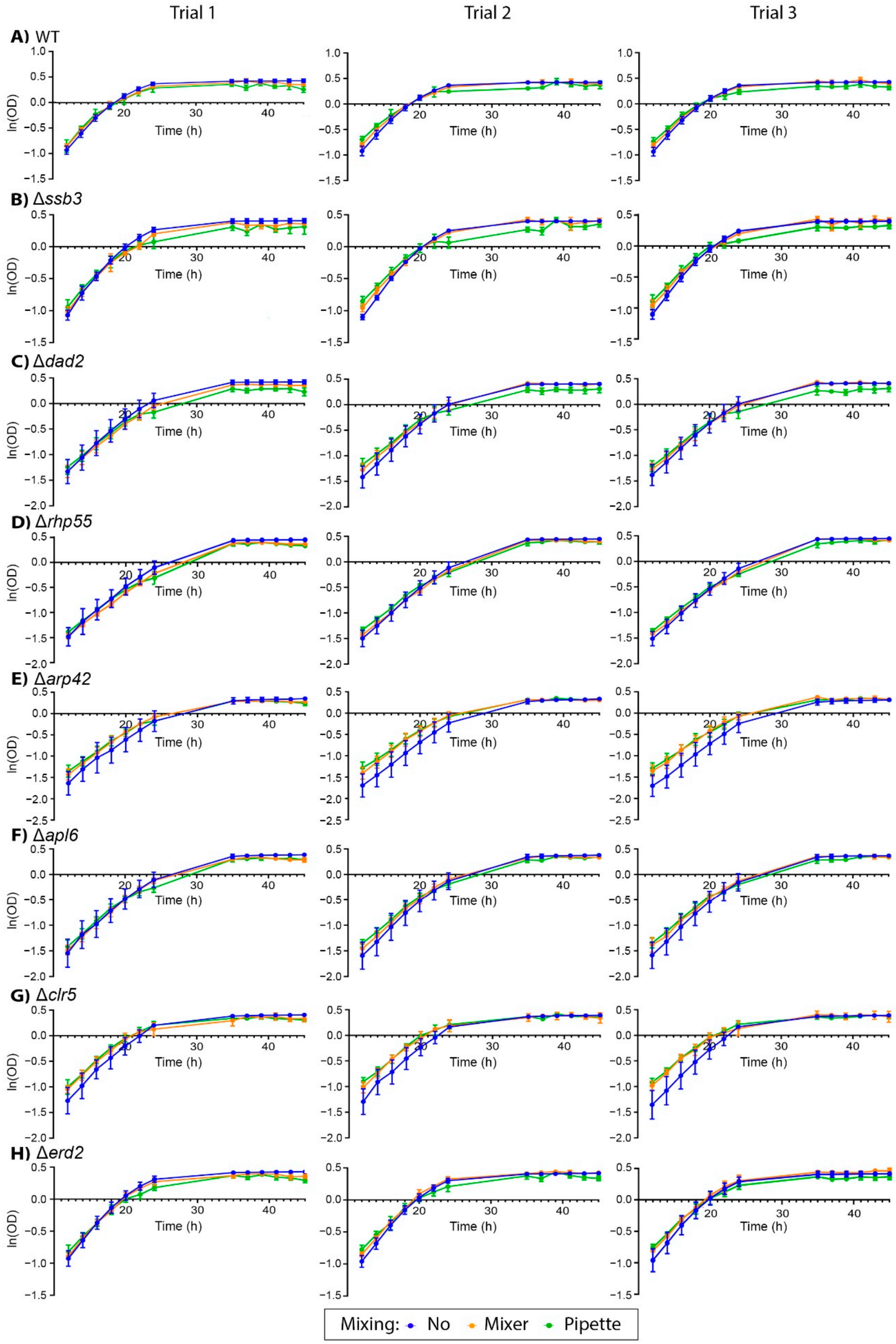

Supplementary Figure S4. Growth curves of WT and mutants over time. Graph of ln(OD) vs time (hour) was plotted for all three trials of (**A**) WT and knockout mutants (Δ) of (**B**) *ssb3*, (**C**) *dad2*, (**D**)

rhp55, (**E**) *arp42*, (**F**) *apl6*, (**G**) *clr5*, and (**H**) *erd2*. Error bars represent mean ± SD, *N* = 9. Blue line, no mixing; Orange line, mixing with mixer; Green line, mixing with pipette. The average ln(OD) vs time of all three trials was shown in Figure 2.

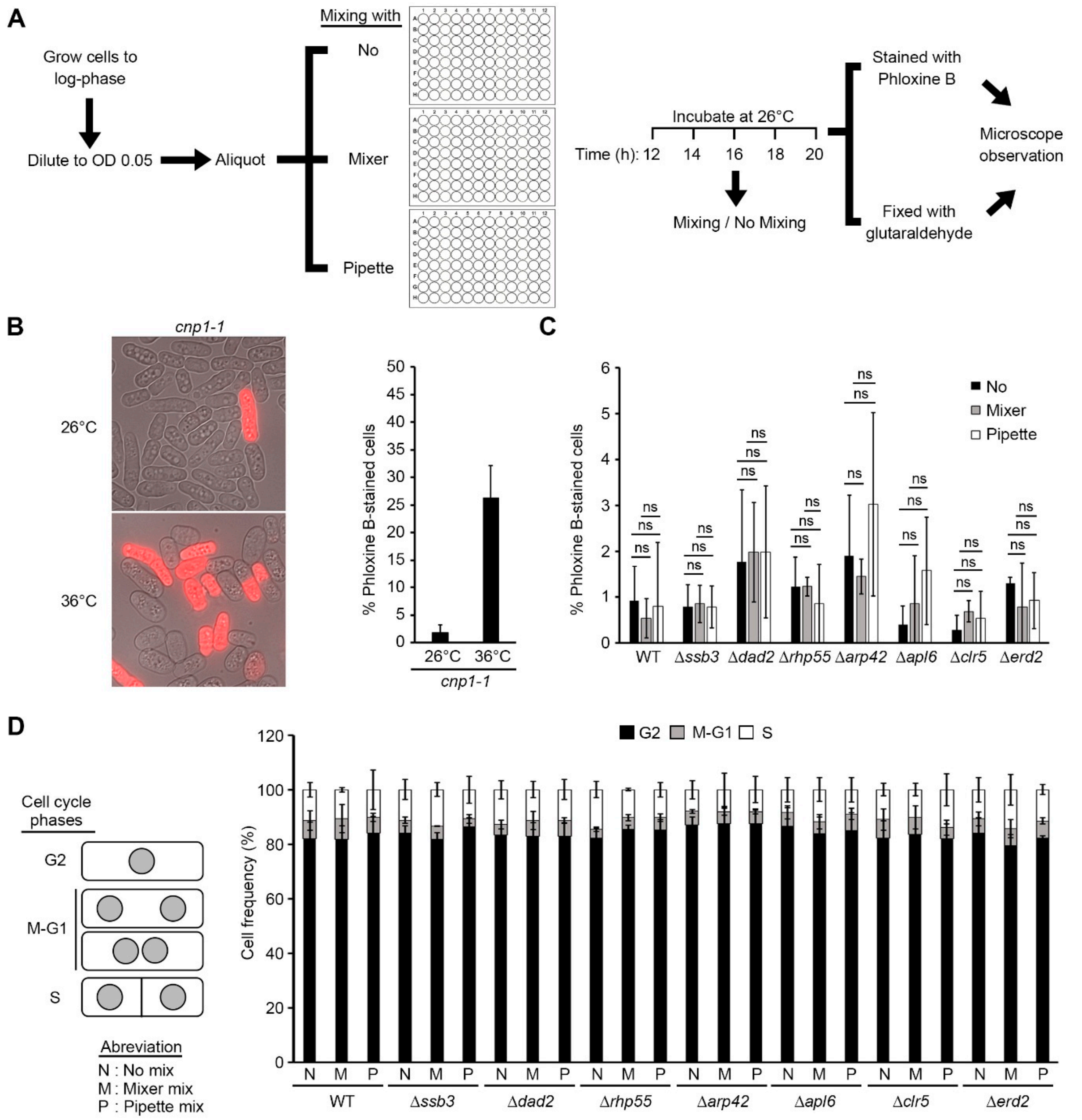

Supplementary Figure S5. Mixing by mixer and pipetting does not affect cell biology. (**A**) Schematic diagram of experiment done. Mixing was done at 12-20 h, once every two hours which corresponds to the exponential phase mentioned in the main text. Cells was stained with phloxine B for 1 h or glutaraldehyde fixed for 15 mins before washed with PBS and observed under microscope. (**B**)

Temperature sensitive Cnp1 mutant strain, *cnp1-1*, was used as control. Cells stained red represent cells that lost viability hence allowing the phloxine B dye to enter. Representative images shown were the overlapped images of DIC and TRITC channels. Right, quantification of red-stained *cnp1-1* in 26 °C and 36 °C. *N*=3. Bars and error bars represent the mean ± S.D., respectively. (**C**) Quantification of phloxine B-stained cells in WT and various mutants in no mixing (No, black bar), mixer mixed (Mixer, gray bar) and pipette mixed (Pipette, white bar) samples. *N*=3. Bars and error bars represent the mean ± S.D., respectively. ns, not significant. (**D**) Quantification of cells with a single nucleus (black, G2 phase), two nuclei without septum (gray, M to G1 phase), and binucleated cell with septum (white, S phase) in WT and various mutants under different mixing conditions. N, unmixed; M, mixer mixed; P, pipette mixed. *N*=3. Bars and error bars represent the mean ± S.D., respectively. At least 200 cells were counted in each experiment of panel B-D.

Supplementary Video S1: Video clip showing how mixing was performed using the mixer.
